# Bioinspired Materials for Water Purification

**DOI:** 10.3390/ma9060447

**Published:** 2016-06-03

**Authors:** Alfredo Gonzalez-Perez, Kenneth M. Persson

**Affiliations:** 1 South Sweden Water Supply (Sydvatten AB), Skeppsgatan 19, Malmö SE-21119, Sweden; kenneth.m.persson@sydvatten.se or kenneth_m.persson@tvrl.lth.se; 2 Sweden Water Research AB, Ideon Science Park, Scheelevägen 15, Lund SE-22370, Sweden; 3 Department of Water Resources Engineering, Lund University, P.O. Box 118, Lund SE-22100, Sweden; 4 Membrane Biophysics, Niels Bohr Institute, Blegdamsvej 17, Copenhagen 2100, Denmark

**Keywords:** water purification, environmental remediation, bioinspired materials, block copolymers, self-assembly, biomimetic membranes, mesoporous materials, liquid crystals, advanced oxidation processes, artificial biofilms, bioreactors

## Abstract

Water scarcity issues associated with inadequate access to clean water and sanitation is a ubiquitous problem occurring globally. Addressing future challenges will require a combination of new technological development in water purification and environmental remediation technology with suitable conservation policies. In this scenario, new bioinspired materials will play a pivotal role in the development of more efficient and environmentally friendly solutions. The role of amphiphilic self-assembly on the fabrication of new biomimetic membranes for membrane separation like reverse osmosis is emphasized. Mesoporous support materials for semiconductor growth in the photocatalytic degradation of pollutants and new carriers for immobilization of bacteria in bioreactors are used in the removal and processing of different kind of water pollutants like heavy metals. Obstacles to improve and optimize the fabrication as well as a better understanding of their performance in small-scale and pilot purification systems need to be addressed. However, it is expected that these new biomimetic materials will find their way into the current water purification technologies to improve their purification/removal performance in a cost-effective and environmentally friendly way.

## 1. Introduction

About 2.5% of the total amount of water on earth is freshwater and of this only about 0.007% is available for human consumption. Out of all global freshwater withdrawal, about 70% is used in agriculture, 20% for industrial (including energy) use, and 10% for water-related needs of households, institutions, municipal systems, and small-medium size industries [1,2]. The various uses of freshwater require different degrees of processing/purification, and only in very rare cases can water be used directly from the reservoir where is stored. Freshwater stored in lakes, rivers, and groundwater reservoirs are the main source for human consumption, while glaciers, ice caps, and water stored in permafrost are less accessible and therefore less used.

Unfortunately, freshwater is not uniformly distributed around the globe and some regions have relatively abundant resources of water, while others face drought and pollution problems. Water scarcity is a serious global issue, and, according to the United Nations World Water Development Report 2016, it is estimated that 1.8 billion people will live in areas with water scarcity by 2025, while two thirds of the world’s population will be living in water-stressed regions [1]. It is known that freshwater withdrawals have increased globally by about 1% per year since the 1980s, mainly due to growing demand in developing countries. Additionally, water scarcity directly affects the job market because 78% of jobs constituting the global workforce are dependent on water.

Industry and manufacturing play their part, accounting for approximately 4% of global water withdrawals. It has been predicted that, by 2050, manufacturing alone could increase its water use by 400%. Because of the current situation and increasing prospective needs of water, monumental challenges in conservation, management, and distribution are ahead [1,3].

It is expected that advances in water purification technology will play an increasingly relevant role for meeting the current and future water needs for agricultural, industrial, and domestic use [4]. Water is being processed/purified by physical, chemical, and/or biological methods, where the physicochemical properties of new materials used within those processes, as well as their ability to add functionality for the specific removal task, will drive future advances in water purification and processing techniques. A wide variety of organic and inorganic materials are being used at different steps of the purification process of typical surface water treatment plants: clarification, filtration, and disinfection [5,6,7].

Development of new biomimetic or bioinspired materials will play a pivotal role in future water purification technologies [8]. Until recently, we could fairly claim that most of the technological development in many increasingly relevant water research areas, like membrane separation technologies, where performed using a heuristic approach. The most recent advances in membrane separation rely on ideas and strategies initiated in the 1970s that evolved by trial-and-error into our current membrane separation technologies—most of which use polymers, [9,10]. Future materials are being developed with a more rational approach, and in many cases are inspired by naturally existing materials form biological and non-biological origin. Many of these new materials are based on the well-known self-assembly properties of amphiphilic molecules, such as block copolymers and surfactants, that are ubiquitous in living organisms. Because the science behind self–assembly is fairly well understood, this will allow a more controllable tuning in the development of new materials, rendering the current heuristic strategy a thing of the past.

In this review, we will focus on the impact of self-assembly on the development of new bioinspired materials and their role in future water purification technologies. Those materials can be built up from their fundamental amphiphilic constituents, present in all biological organisms, or from man-made analogues, exploiting their intrinsic self-assembly properties in a fine-tuned fashion. We will stress the recent approaches in biomimetic membrane development, the bioinspired templates for advance oxidation processes, and novel carrier supports for artificial biofilms for bioreactors. The development of new bioinspired materials will continue to play a preponderant role in the current and future water purification and environmental remediation technologies.

## 2. Ampiphiles: Building Structure via Self-Assembly

Molecules that contain structural hydrophobic and hydrophilic motifs can undergo aggregation under the right thermodynamic conditions of concentration, temperature, and pressure. Those molecules are called amphiphiles, and their self-assembly properties are driven by reversible non-covalent interactions. The chemical architecture of the amphiphiles, the individual building blocks, can be tuned to generate supramolecular materials where multiple interactions can determine their final properties, including morphology, mechanical strength, and responsiveness, among others [11,12].

The control of the material properties is intimately related to interactions that generate the material such as hydrogen bonding, hydrophobic interactions, π–π stacking, host–guest interactions, and metal ligand coordination. A comprehensive summary of all possible interactions is out of the scope of the current review and can be found elsewhere [13,14,15,16]. The type of self-assembled structure can be predicted based on geometrical constraints of the amphiphile or curvature parameters of a membrane formed with amphiphiles.

**Figure 1:**
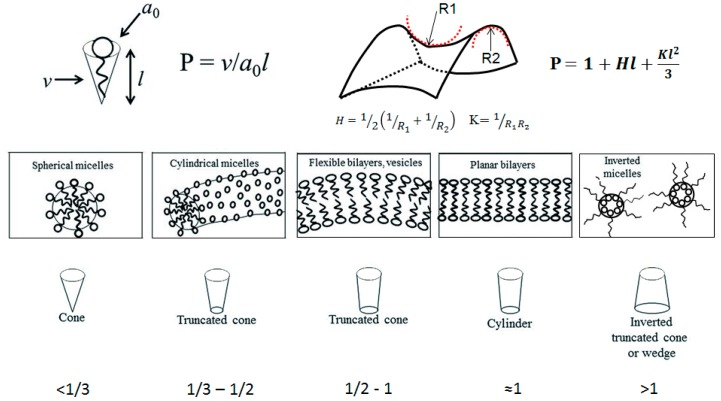
Above: the figure shows the packing parameter *versus* the geometrical constraints of a typical amphiphile (left) and the correlation with the curvature parameters (right); Below: different self-assembled structures with their corresponding packing parameter values.

The packing parameter model is based on geometrical constraints and was introduced by Israelachvili *et al.* in 1976 [15]. In their model, the packing parameter in a typical amphiphile is correlated with the volume occupied by the hydrophobic motif (hydrocarbon chain), the surface area occupied by the head group, and the length of the hydrophobic motif, as is shown in Figure 1. Depending on the values of the packing parameter, P, we can get different structures such as micelles (spherical or cylindrical), bilayers and vesicles, planar bilayers, and the inverted analogues. It is also possible to predict the resulting structures by looking at the elastic free energy that is associated with the curvature of a surface. In this case, it is possible to correlate the packing parameter with the mean curvature, H, and the Gaussian curvature, K. The elastic free energy density is associated with the curvature of the surface. A description of the different self-assembled architectures and key parameters of both approaches, as well as the structures associated with specific P values, are summarized in Figure 1.

A wide variety of supramolecular architectures can be obtained; spherical and wormlike micelles, vesicles, lamellar, cubic and hexagonal phases, and more exotic bi-continuous phases. These self-organized systems usually serve as structure-guiding media for the development of mesoporous materials for different applications [17,18,19].

**Figure 2:**
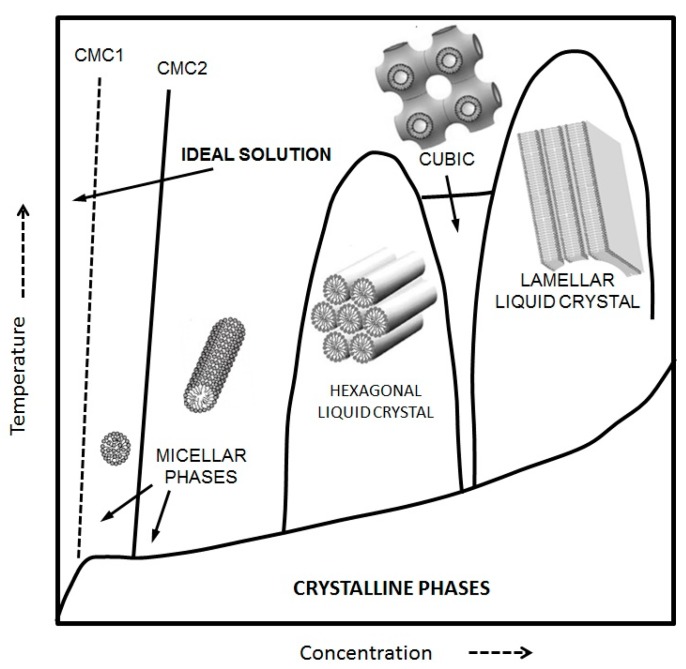
Typical phase diagram showing the different self-assembled structures as a function of concentration and temperature for a general amphiphile.

However, both curvature and geometrical constraints associated with the packing parameter are not unique factors defining the kind of self-assembled structures we know of. Thermodynamic parameters such as pressure, temperature, and concentration modulate the phase behavior of the different structures. Phase transitions between self-assembled structures do happen when temperature and concentration shifts occur. A typical phase diagram for an amphiphilic compound can be seen in Figure 2.

Supramolecular structures are commonly built with block copolymers, and the literature about the formation of these soft materials is abundant. Excellent summaries have been published in recent years by Hamley [20,21,22]. The physics behind the phase behavior and the mechanism for control of the phase transitions are well-known [23,24]. Polymer self-assembly represents an excellent approach for new biomimetic nanostructures for liquid filtration membranes [25]. Mesoporous materials can be fabricated by using lyotropic liquid crystals as a template [7]. These materials are useful as membrane supports for water separation techniques or can alternatively be used to create supports for bacterial immobilization or semiconductor growth for bioreactor processing and the photocatalytic degradation of water pollutants, respectively.

## 3. Biomimetic Membranes

Membranes have been used for decades in separation techniques and are currently widely used in water purification processes. We can classify the membranes in two main classes: isotopic and anisotropic, that is, homogenous and heterogeneous in composition, respectively. There are a wide variety of membranes belonging to one of these two main categories. Among the anisotropic ones, phase-separation membranes and composite membranes such as thin-film composite membranes (TFC) are widely used in current reverse osmosis (RO) purification. A review of the progress in membrane science and technologies for water purification was recently published by Lee *et al*. [9].

Although membranes can be prepared from inorganic materials, the most prevalent materials used in commercial membranes are synthetic polymers with differences in preparation methodology that result in different ending pore sizes. It is increasingly common that the development of hybrid membranes combines both organic, mostly polymeric, materials with inorganic ones such as metal oxides in the form of composites [26].

Membranes can be classified according to their filtration properties determined by their maximum pore size as follows: reverse osmosis (RO), 1 nm; nanofiltration (NF), 2 nm; ultra filtration (UF), 100 nm; micro filtration (MF), 10 µm; and particle filtration, 1000 µm. The common goals for all membranes to be optimal are (a) high flux, permeation, and rejection; (b) mechanical, chemical, thermal, and temporal stability; (c) system design, including processability into large scale; (d) cost-effectiveness; and (e) anti-fouling. This can be seen as a general design guide, as was suggested by Lee *et al.* [9]. Desalination is the more recurrent technology on arid regions relaying mainly in the use of polyamide-based membranes. Alternative membrane methods that improve the performance of the current ones are likely to emerge in the near future. The use of biomimetic membranes is an emerging technology with potential uses in separation techniques [27].

Biomimetic block copolymer membranes have been used for functional membrane protein reconstitution, mimicking biological membranes [28]. In particular, aquaporin Z (AQP Z), a naturally occurring water channel, was incorporated in a functional form in a triblock copolymer, poly(2-methyl-2-oxazoline)-*block*-poly(dimethylsilozane)-*block*-poly(2-methyl-2-oxazoline), showing a better water permeability than polyamide composite membranes [29]. Large block copolymer membrane arrays were prepared with functional gramicidin A as a probe of concept, demonstrating that large membrane arrays could be keep stable for long periods of time [30]. However, the science behind the reconstitution of membrane proteins into block copolymer membranes is still in its infancy and is being continually updated since the pioneering works of Meier *et al.* [31,32,33,34]. The use of aquaporins in membrane purification together with carbon nanotubes [35] are excellent choices for water filtration due to their high water flux. Unfortunately, membranes prepared containing aquaporins or carbon nanotubes are very expensive; hence, both membranes are still far from representing a cost-effective alternative to polyamide composite membranes.

**Figure 3:**
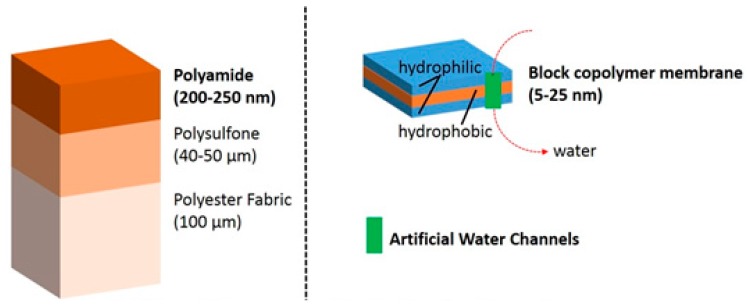
Scheme showing membrane structure a characteristic sizes. A typical composite membrane with the three layers based on polyamide, polysulfone, and polyester (**left**). A biomimetic block copolymer membrane with an artificial water channel incorporated as a functional motif (**right**).

The works of Montemagno and Meier in the US and Switzerland, respectively, have opened the door to the use of block copolymers as a support for functional reconstitution of membrane proteins [36]. In order to put in perspective the differences between conventional composite polyamide reverse osmosis membranes and new biomimetic polymeric membranes with artificial water channels, we show the main scale and composition differences in Figure 3.

The number of artificial water channels, designed to selectively transport water in an efficient manner through bilayer membranes, is very limited and relies mainly on the use of lipid membranes [37]. Dendritic dipeptides, oriented dipolar water wires based on ureidomidazone, and hydrazide-functionalized pillar(5)arene are among the artificial water channels with demonstrated functionality. In a more recent publication by Shen *et al.* [38], an artificial peptide-appended pillar(5)arene was incorporated into lipid membranes showing a flux of 3.5(±1.0) × 10^8^ water molecules per second comparable with the values reported by aquaporin-based membranes. The information about the general performance of new biomimetic membranes with aquaporins or other water channels is very limited because this research approach is still in its infancy and more systematic work is needed. Using water permeability on proteoliposomes containing AQP Z, Kumar *et al.* [39] estimated an expected membrane permeability of 167 μm∙s^−1^∙bar^−1^. The performance data on water permeability, NaCl rejection, membrane area, and maximal external pressure for different biomimetic membranes has been recently summarized by Tang *et al.* [40]. There are new, interesting opportunities here, such as combining artificial water channels with block copolymer membranes for the creation of new membranes to be used in reverse osmosis processes.

## 4. Mesoporous Materials Templates for Advanced Oxidation Processes

**Figure 4:**
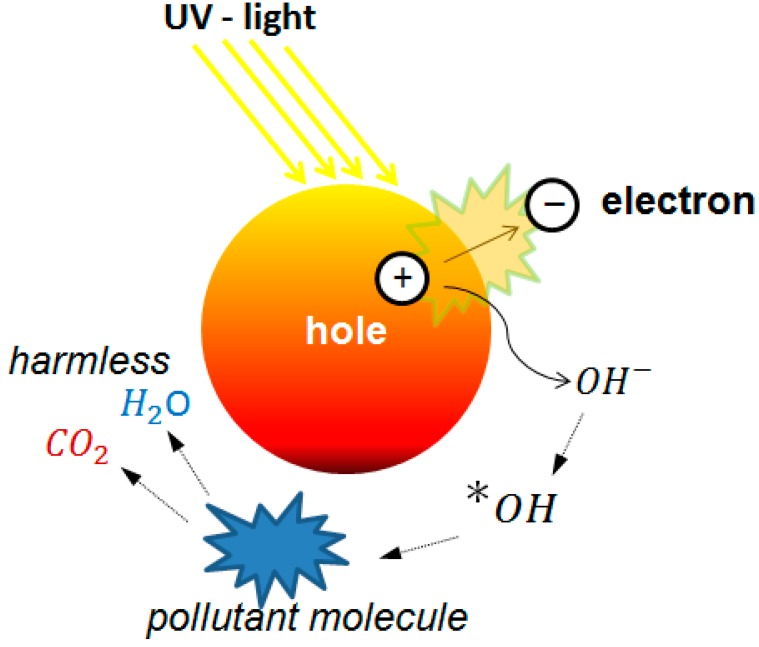
Scheme of the mechanism of electron-hole formation in a semiconductor upon irradiation of UV, and the breakdown of pollutants.

Mesoporous materials have been used as a templated for a wide variety of applications serving as a support and guide for the bottom-up formation of new materials. This approach is especially useful in advanced oxidation that uses ultraviolet (UV) light to activate a semiconductor generating reactive species. Water and air pollutant molecules can be degraded using this approach [41]. In particular, the interaction of UV light with TiO_2_ has been widely investigated, as has the formation of reactive radical species that can readily attack organic chemicals in water. These processes have received increasing attention for environmental remediation due to the environmentally benign properties of TiO_2_ and the elimination of chemical additives [42,43]. Photocatalysis using TiO_2_ have great advantages for their efficiency, stability, and low production cost, although other photocatalysts have been proposed and are a subject of very active research. A scheme of the mechanism of action is shown in Figure 4.

In the past, much research has been carried out into a slurry system (suspension of fine powdered TiO_2_). However, the post-treatment removal of TiO_2_ results in the filtration and resuspension of photocatalyst powder is costly. In order to avoid this step, photocatalyst particles have been used to coat a variety of surfaces, such as glass, silica gel, metal, ceramics, polymer, thin films, fibers, zeolite, alumina clays, activated carbon, cellulose, reactor walls, and many others. An increasingly interesting approach relies on the use of biomimetic mesoporous materials able to display large surface areas in a small volume with a suitable porosity that is necessary to allow a good water flow through them. 

A mesoporous materials with a large surface area and pore size can be obtained using bi-continuous liquid crystals (cubic and sponge phases based on amphiphilic self-assembly) as a template to cast TiO_2_ material in a crystalline anatase phase or a combination of anatase and rutile phases [44]. This material can be used for photocatalytic degradation of organic molecules in water using UV radiation to drive the surface reaction at a high water flow rate. Specifically, the hexagonal phase (H_1_) and lamellar (L_α_) have been successfully used as a template for TiO_2_ crystallization in the form of nanoparticles. However, it is possible to use a cubic and sponge phases (L_3_) as a basic template with the same purpose. The L_3_ phase has a large surface area, is well connected, and displays a low viscosity comparable to that of pure water. This property allows the fast diffusion of the precursors needed for TiO_2_ crystallization without the disruption of the crystalline phase.

The sol-gel synthesis method is widely used to crystallize TiO_2_ on surfactant-based structures such as micelles. However, without special care for the crystallization process, we obtain a polymorph phase composed of different TiO_2_ phases in an uncontrolled manner. This results in poor photoactivity and, by extension, a low performance material. The TiO_2_ anatase crystalline phase can give higher photoactivity than rutile or brookite phases, and there are several available methodologies (protocols) that allow for the obtainment of a pure anatase phase. TiO_2_ nanoparticles can be prepared using the sol-gel method as well as other methodologies to be used in photocatalytic degradation of organic molecules. However, there is an increasing concern about the environmental impact of nano-size materials, and it is likely that more studies will appear in the near future showing adverse effects for the environment and human health. Hence, there is an urgent need to develop new materials that display more environmentally safe characteristics and, at the same time, take advantage of the properties that emerge from nano-size dimensions, such as the large surface area enclosed in a small volume. A high specific surface area is a key parameter to the performance of catalysts, providing enough active sites for optimal catalytic performance. 

A common way to obtain large surface areas for catalysis is by growing the photocatalyst in nanoparticles such as gold [45]. Nanoparticles have been extensively investigated as a suitable support for several oxides like TiO_2_, Fe_2_O_3_, or CeO_2_. A recent review of Wu *et al.* summarized the development of novel mesoporous silica-based gold catalysts [45]. However, nanoparticle-based photocatalysts are difficult to confine, and there is an increasing concern about the environmental safety of TiO_2_ nanoparticles [46,47,48]. This has pushed some research towards the use of porous materials as a support to grow TiO_2_.

Ordered mesoporous materials have found their way into different applications for new catalytic processes. One early review was written by Taguchi and Schuth [49]. The different methods for synthesis of functional mesoporous materials allowed diversification in applications. A short review was published by Fryxell *et al.* [50]. Mesoporous oxides can now be prepared in a wide variety of procedures that are summarized elsewhere [18,19]. In particular, gyroid structures present a large connected surface area that is perfectly suited for photocatalyst purposes in the water business. Gyroid-structured functional materials are of special interest [51,52]. The self-assembly of degradable block copolymers can be used as a template for the synthesis of various mesoporous materials and, in particular, serve as a spendable substrate for the formation of TiO_2_ or other oxide semiconductors to be used in photocatalysis for purification and pollutant removal in water.

Interestingly, the analogous block copolymers to those used for biomimetic membrane formation can be used at higher concentrations to form liquid crystalline structures that can serve as a template for the creation of new mesoporous materials. There are still challenges concerning the formation of TiO_2_ on the right phase or with the right combination of phases for an optimal photocatalytic efficiency. In any case, there is no doubt that new biocontinuous-based structures will find their way into the advance oxidation process for water purification.

## 5. Artificial Biofilm Carriers for Bioreactors

Among the bioinspired materials in water purification and environmental remediation are those that involve the direct use of microorganisms for the breakdown or the transformation, in a controllable manner, of poisonous and harmful pollutants into non-toxic substances. Natural bioremediation using aquatic plants, animals, and microorganisms in recreated environments, such as constructed wetlands, is a widely spread methodology that has shown excellent results [53]. However, this approach has substantial limitations and requires physical space close to the polluted water. Microorganisms like bacteria and archaea can be used under controlled environments in bioreactors to perform the breakdown or transformation of pollutants in a more portable manner. Abundant literature exists about bacterial performance in bioreactors using different bacterial strands in planktonic form. In recent years, an increasing interest in the use of artificial biofilms has been motivated by the interest in improving the survival rate of the bacterial colony and the increase in the transformation rate of the pollutants intended to be removed or processed. 

**Figure 5:**
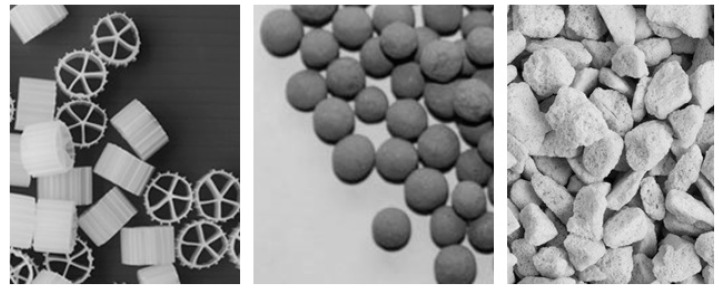
Examples of different types of carriers. From left to right, *Kaldnes MiljØteknologi* (KMT), porous ceramsite, and recycled porous glass.

Biofilms can be grown into different supports (carriers) not only from bacterial and archaeal species that naturally form biofilms, but also from techniques that are well developed to artificially attach planktonic species to different carriers. Immobilization of microorganisms substantially influences their survival rate, as has been demonstrated in fermentation technology [54] Because biofilm formation is essentially a surface phenomenon, a key objective is to maximize the surface area of the carrier available for immobilization and at the same time obtain a uniform distribution of the artificial bacterial film over the available surface. Examples of biofilm carriers are shown in Figure 5.

The most advanced bioreactor techniques and designs focus on the removal of nitrogen and phosphorous, which is a common problem derived from agricultural practices. Biofilm reactors are continuously being developed for a wide variety of water-related applications with different removal capabilities [55,56]. Considerable effort has being paid to developing mathematical models for anaerobic reactors in order to optimize their design, design the process control systems used in their operation, and enhance their operational efficiency. A critical review of the different mathematical models available for these reactors has been done by Saravanan *et al.* [57].

Most recently, there has been a growing interest in the development of biofilm-based bioreactors for the processing of different wastewaters for the recovery of metals, rare-earth minerals, radioactive isotopes, *etc.* [58]. However, the impact of those technologies is still very limited to fundamental research basic prototyping. Further development will be needed to better understand and control the mechanism of processing and to ensure a high recovery yield of the byproducts generated by the artificial biofilm. Hybrid biofilm-activated sludge has been successfully used for the removal of micropollutants from water. The methodology uses suspended/attached growth of bacteria forming a biofilm in a carrier made of a porous material in combination with activated sludge [59]. The carriers can be designed in different ways to adapt to different applications; they could be fixed like a polymeric membrane or mobile floating on the water reservoir. Microorganism immobilization is currently being used successfully in fermentation techniques, leading to better production yields than the planktonic analogues. More recently, the environmental removal of estrogen was achieved with different strands of bacteria able to degrade estrogenic substances. The bacterial strands where immobilized into a porous structure that enhanced their viability [60]. A cross-linked polymer matrix as a support to immobilize specific bacterial strands was used for the removal of heavy metals from water. [61]. The recent methodological advances for heavy metal removal from waste water have been summarized by Fu and Wang [62]. Immobilized bacteria have been used in more specific applications for rare-earth separation [63]. Further development of new carriers and support mesoporous materials together with a better understanding of the bacterial ecology on the artificial biofilm will help to develop the research field and provide new functional materials for water remediation.

It is expected that the immobilization of new bacterial species with specific removal capabilities for pollutants, in combination with the use of optimal mesoporous carriers, will help this water remediation approach to gain more acceptance in the water and wastewater processing businesses and expand into other industries with similar characteristics. The development of more portable and versatile bioreactors will help to move this technological approach towards a state of maturity with a wide business potential in wastewater treatment, acid mine drainage, stream and/or lake bioremediation, among others.

## 6. Summary and Future Perspectives

Biomimetic materials have been developed for a long time, expanding their range of action to include more applications. In the water business, new biomimetic block copolymer membranes with water permeation motifs, the biocontinuous liquid crystalline templates for TiO_2_ deposition for advance oxidation processes, and the new artificial biofilms under controlled environments for pollutant removal are among the most promising biomimetic approaches for future water purification and environmental remediation. 

Challenges are ahead to develop suitable applications of those technologies into economically viable products that could enhance or even displace current water purification technologies. Within the biomimetic polymeric membranes with functional water pores, the density and proper functionality of the permeation motifs remains to be optimized. Aquaporins have been demonstrated to work in an analogous manner to those present in biological membranes. However, the correct orientation of the protein with high density incorporation in an economically viable is still a subject of concern. Some advances have been done by the development of a large production of the protein and its incorporation into a composite material. Other functional pores like carbon nanotubes need to address the problem of acquiring a large optimal nanotube length and some safety concerns. 

The development of new photocatalytic reactors for polluter removal will certainly take advantage from the liquid crystalline structures as a template for the growth of TiO_2_ and other photocatalysts. Challenges remain ahead on the precise control of the TiO_2_ phases and the optimization of the anatase–rutile composition. Those problems are not easy to address in structures that are thermodynamically stable and can be disrupted upon small changes in temperature and composition. The use of cross-linkable motifs could help to fix natural instability problems and allow the development of functional cost-effective photocatalysts. This approach is likely to be preferred in the market because of the inherently expensive methods for TiO_2_ formation via vapor deposition. 

Looking at artificial biofilm deposition for bioreactors used in pollutant removal will face challenges for the upscale of bioreactors to be used in high-flow situations and to address the long-term need for biofilm formation. Therefore, much more research is needed to improve our understanding of those mesoporous biomimetic materials and optimize their usage for practical applications in water purification and related remediation technologies.

## Abbreviations

The following abbreviations are used in this manuscript:

UV	ultraviolet light
TFC	thin-film composite membranes
AQP Z	aquaporin Z
KMT	*Kaldnes MiljØteknologi*
H1	hexagonal phase (H_1_)
Lα	lamellar phase (L_α_)
L3	sponge phase (L_3_)
TiO_2_	titanium dioxide
RO	reverse osmosis
NF	nanofiltration
UF	ultra filtration
MF	micro filtration

## References

[1] United Nations World Water Assessment (WWAP) (2016). The United Nations World Water Development Report 2016.

[2] Gleick P.H. (1993). Water in Crisis: A Guide to the World’s Fresh Water Resources.

[3] Kulshreshtha S.N. (1998). A global outlook for water resources to the year 2025. Water Resour. Manag..

[4] Shannon M.A., Bohn P.W., Elimelech M., Georgiadis J.G., Mariñas B.J., Mayes A.M. (2008). Science and technology for water purification in the coming decades. Nature.

[5] The American Water Works Association (AWWA), The American Society of Civil Engineers (ASCE) (2012). Water Treatment Plant Design.

[6] Kawamura S. (2000). Integrated Design and Operation of Water Treatment Facilities.

[7] Edzwald J., The American Water Works Association (AWWA) (2010). Water Quality & Treatment: A Handbook on Drinking Water.

[8] Yunus I.S., Harwin, Kurniawan A., Adityawarman D., Indarto A. (2012). Nanotechnologies in water and air pollution treatment. Environ. Technol. Rev..

[9] Lee A., Elam J.W., Darling S.B. (2016). Membrane materials for water purification: Design, development, and application. Environ. Sci. Water Res. Technol..

[10] Geise G.M., Lee H.-S., Miller D.J., Freeman B.D., McGrath J.E., Paul D.R. (2010). Water purification by membranes: The role of polymer science. J. Polym. Sci. Part B Polym. Phys..

[11] Zana R., Zana R. (2005). Dynamics of Surfactant Self-Assemblies: Micelles, Microemulsions, Vesicles and Lyotropic Phases.

[12] Garti N. (2012). Self-Assembled Supramolecular Architectures: Lyotropic Liquid Crystals.

[13] Nagarajan R. (2002). Molecular packing parameter and surfactant Self-Assembly: The Neglected Role of the Surfactant Tail. Langmuir.

[14] Hyde S., Blum Z., Landh T., Lidin S., Ninham B.W., Andersson S., Larsson K. (1996). The Language of Shape: The Role of Curvature in Condensed Matter: Physics, Chemistry and Biology.

[15] Israelachvili J.N., Mitchell D.J., Ninham B.W. (1976). Theory of self-assembly of hydrocarbon Amphiphiles into Micelles and Bilayers. J. Chem. Soc. Trans. I.

[16] Israelachvili J.N. (2011). Intermolecular and Surface Forces.

[17] Meier W. (1999). Nanostructure synthesis using surfactants and copolymers. Curr. Opin. Colloid Interface Sci..

[18] Ciesla U., Schüth F. (1999). Ordered mesoporous materials. Microporous Mesoporous Mater..

[19] Meynen V., Cool P., Vansant E.F. (2009). Verified syntheses of mesoporous materials. Microporous Mesoporous Mater..

[20] Hamley I.W. (2003). Nanotechnology with soft materials. Angew. Chem. Int. Ed..

[21] Hamley I.W. (2003). Nanostructure fabrication using block copolymers. Nanotechnology.

[22] Hamley I.W. (2005). Nanoshells and nanotubes from block copolymers. Soft Matter.

[23] Hamley I.W. (1998). The Physics of Block Copolymers.

[24] Hamley I.W. (2005). Block Copolymers in Solution: Fundamentals and Applications.

[25] Asatekin A., Vannucci C. (2015). Self-assembled polymer nanostructures for Liquid Filtration Membranes: A Review. Nanosci. Nanotechnol. Lett..

[26] Montemor M. (2015). Smart Composite Coatings and Membranes: Transport, Structural, Environmental and Energy Applications.

[27] Hélix-Nielsen C. (2012). Biomimetic Membranes for Sensor and Separation Applications.

[28] Zhang X., Tanner P., Graff A., Palivan C.G., Meier W. (2012). Mimicking the cell membrane with block copolymer membranes. J. Polym. Sci. Part A-Polym. Chem..

[29] Kumar M., Habel J.E.O., Shen Y., Meier W.P., Walz T. (2012). High-density reconstitution of functional Water Channels into Vesicular and Planar Block Copolymer Membranes. J. Am. Chem. Soc..

[30] González-Pérez A., Stibius K.B., Vissing T., Nielsen C.H., Mouritsen O.G. (2009). Biomimetic triblock copolymer membrane Arrays: A Stable Template for Functional Membrane Proteins. Langmuir.

[31] Stoenescu R., Meier W. (2004). Asymmetric membranes from amphiphilic ABC triblock copolymers. Mol. Cryst. Liq. Cryst..

[32] Nardin C., Widmer J., Winterhalter M., Meier W. (2001). Amphiphilic block copolymer nanocontainers as bioreactors. Eur. Phys. J. E.

[33] Kita-Tokarczyk K., Grumelard J., Haefele T., Meier W. (2005). Block copolymer vesicles—Using concepts from polymer chemistry to mimic biomembranes. Polymer.

[34] Hua D., Kuang L., Liang H. (2011). Self-directed reconstitution of proteorhodopsin with Amphiphilic Block Copolymers Induces the Formation of Hierarchically Ordered Proteopolymer Membrane Arrays. J. Am. Chem. Soc..

[35] Hinds B.J., Chopra N., Rantell T., Andrews R., Gavalas V., Bachas L.G. (2004). Aligned multiwalled carbon nanotube membranes. Science.

[36] Ruso J.M., Piñeiro Á. (2013). Proteins in Solution and at Interfaces.

[37] Barboiu M. (2012). Artificial water channels. Angew. Chem. Int. Ed..

[38] Shen Y., Si W., Erbakan M., Decker K., de Zorzi R., Saboe P.O., Kang Y.J., Majd S., Butler P.J., Walz T. (2015). Highly permeable artificial water channels that can self-assemble into two-dimensional arrays. Proc. Natl. Acad. Sci. USA.

[39] Kumar M., Grzelakowski M., Zilles J., Clark M., Meier W. (2007). Highly permeable polymeric membranes based on the incorporation of the functional water channel protein Aquaporin Z. Proc. Natl. Acad. Sci. USA.

[40] Tang C.Y., Zhao Y., Wang R., Hélix-Nielsen C., Fane A.G. (2013). Desalination by biomimetic aquaporin membranes: Review of status and prospects. Desalination.

[41] Augugliaro V., Loddo V., Pagliaro M., Palmisano G., Palmisano L. (2010). Clean by Light Irradiation.

[42] Fujishima A., Rao T.N., Tryk D.A. (2000). Titanium dioxide photocatalysis. J. Photochem. Photobiol. C Photochem. Rev..

[43] Huber B., Brodyanski A., Scheib M., Orendorz A., Ziegler C., Gnaser H. (2005). Nanocrystalline anatase TiO_2_ thin films: Preparation and crystallite size-dependent properties. Thin Solid Films.

[44] Luttrell T., Halpegamage S., Tao J., Kramer A., Sutter E., Batzill M. (2014). Why is anatase a better photocatalyst than rutile?–Model studies on epitaxial TiO_2_ films. Sci. Rep..

[45] Wu H., Pantaleo G., Venezia A., Liotta L. (2013). Mesoporous silica based gold catalysts: Novel synthesis and Application in Catalytic Oxidation of CO and Volatile Organic Compounds (VOCs). Catalysts.

[46] Sharma V.K. (2009). Aggregation and toxicity of titanium dioxide nanoparticles in aquatic environment–A review. J. Environ. Sci. Health A Tox. Hazard. Subst. Environ. Eng..

[47] Schilling K., Bradford B., Castelli D., Dufour E., Nash J.F., Pape W., Schulte S., Tooley I., van den Bosch J., Schellauf F. (2010). Human safety review of “nano” titanium dioxide and zinc oxide. Photochem. Photobiol. Sci..

[48] Kahru A., Dubourguier H.-C. (2010). From ecotoxicology to nanoecotoxicology. Toxicology.

[49] Taguchi A., Schüth F. (2005). Ordered mesoporous materials in catalysis. Microporous Mesoporous Mater..

[50] Fryxell G.E. (2006). The synthesis of functional mesoporous materials. Inorg. Chem. Commun..

[51] Wu L., Zhang W., Zhang D. (2015). Engineering gyroid-structured functional Materials via Templates Discovered in Nature and in the Lab. Small.

[52] Hsueh H.-Y., Yao C.-T., Ho R.-M. (2015). Well-ordered nanohybrids and nanoporous materials from gyroid block copolymer templates. Chem. Soc. Rev..

[53] Chandra R. (2015). Advances in Biodegradation and Bioremediation of Industrial Waste.

[54] Ramakrishna S.V., Prakasham R.S. (1999). Microbial fermentations with immobilized cells. Curr. Sci..

[55] Muffler K., Roland U. (2014). Productive Biofilms.

[56] Characklis W.G., Marshall K.C. (1990). Biofilm.

[57] Saravanan V., Sreekrishnan T.R. (2006). Modelling anaerobic biofilm reactors—A review. J. Environ. Manag..

[58] Pawłowski L., Gonzales M.A., Dudzińska M.R., Lacy W.J. (1998). Chemistry for the Protection of the Environment 3.

[59] Falås P., Longrée P., la Cour Jansen J., Siegrist H., Hollender J., Joss A. (2013). Micropollutant removal by attached and suspended growth in a hybrid biofilm-activated sludge process. Water Res..

[60] Ma C., Qin D., Sun Q., Zhang F., Liu H., Yu C.-P. (2016). Removal of environmental estrogens by bacterial cell immobilization technique. Chemosphere.

[61] Pires C., Marques A.P.G.C., Guerreiro A., Magan N., Castro P.M.L. (2011). Removal of heavy metals using different polymer matrixes as support for bacterial immobilisation. J. Hazard. Mater..

[62] Fu F., Wang Q. (2011). Removal of heavy metal ions from wastewaters: A review. J. Environ. Manag..

[63] Bonificio W.D., Clarke D.R. (2016). Rare-earth separation using bacteria. Environ. Sci. Technol. Lett..

